# Travel, Treatment Choice, and Survival Among Breast Cancer Patients: A Population-Based Analysis

**DOI:** 10.1089/whr.2020.0094

**Published:** 2021-01-11

**Authors:** Colleen F. Longacre, Hannah T. Neprash, Nathan D. Shippee, Todd M. Tuttle, Beth A. Virnig

**Affiliations:** ^1^ Division of Health Policy and Management, University of Minnesota School of Public Health, Minneapolis, Minnesota, USA.; ^2^ Department of Surgery, University of Minnesota Medical School, Minneapolis, Minnesota, USA.

**Keywords:** breast cancer, patient decision making, radiation treatment, travel distance

## Abstract

***Background:*** Travel distance to care facilities may shape urban–rural cancer survival disparities by creating barriers to specific treatments. Guideline-supported treatment options for women with early stage breast cancer involves considerations of breast conservation and travel burden: Mastectomy requires travel for surgery, whereas breast-conserving surgery (BCS) with adjuvant radiation therapy (RT) requires travel for both surgery and RT. This provides a unique opportunity to evaluate the impact of travel distance on surgical decisions and receipt of guideline-concordant treatment.

***Materials and Methods:*** We included 61,169 women diagnosed with early stage breast cancer between 2004 and 2013 from the Surveillance Epidemiology and End Results (SEER)-Medicare database. Driving distances to the nearest radiation facility were calculated by using Google Maps. We used multivariable regression to model treatment choice as a function of distance to radiation and Cox regression to model survival.

***Results:*** Women living farthest from radiation facilities (>50 miles vs. <10 miles) were more likely to undergo mastectomy versus BCS (odds ratio [OR]: 1.48, 95% confidence interval [CI]: 1.22–1.79). Among only those who underwent BCS, women living farther from radiation facilities were less likely to receive guideline-concordant RT (OR: 1.72, 95% CI: 1.32–2.23). These guideline-discordant women had worse overall (hazards ratio [HR]: 1.50, 95% CI: 1.42–1.57) and breast-cancer specific survival (HR: 1.44, 95% CI: 1.29–1.60).

***Conclusions:*** We report two breast cancer treatments with different clinical and travel implications to show the association between travel distance, treatment decisions, and receipt of guideline-concordant treatment. Differential access to guideline-concordant treatment resulting from excess travel burden among rural patients may contribute to rural–urban survival disparities among cancer patients.

## Introduction

Survival disparities persist between rural and urban cancer patients.^1^ Travel distance to care facilities may contribute to these disparities by creating barriers to accessing specific treatments; however, it is often difficult to disentangle factors impacting patient treatment choices. Women diagnosed with early stage breast cancer have two guideline-supported treatment options: (1) mastectomy and (2) breast-conserving surgery (BCS) followed by radiation therapy (RT).^2–4^ Although overall survival between the two choices is equivalent, BCS+RT is the preferred strategy of the majority of eligible patients^5^ likely because it has been associated with lower complications and costs^6^ and superior quality of life relative to mastectomy.^7^ However, ∼15%–30% of patients who undergo BCS do not receive recommended RT.^5,8–10^ Disparities in RT completion have been associated with age, race, and geography^5^ and have been linked to poorer disease-specific outcomes and overall survival.^11,12^ Little is known about how prevalent incomplete RT (receipt of some RT but not the full recommended course) is among early stage breast cancer patients, although this has also been associated with poorer patient outcomes in some studies.^11,13^ Distance may play a significant role in patient decision making regarding surgery and completion of RT, since RT requires daily travel to radiation facilities for several weeks at a time. The combination of two clinically comparable treatment options with different travel implications provides a unique opportunity to evaluate the impact of travel distance on care decisions and receipt of guideline-concordant treatment.

Available evidence from mostly state-level studies suggests that patients who live farther from radiation facilities may be more likely to choose mastectomy^14–22^ or to not complete recommended radiation after BCS.^14,17,21–23^ In this study, we evaluate the association between travel distance and choice of surgical treatment (mastectomy vs. BCS) and optimal receipt of radiation after BCS among newly diagnosed breast cancer patients in the Medicare population. We then explore the relationship between travel distance, optimal treatment receipt, and survival outcomes.

## Materials and Methods

### Data

Data for this study came from the National Cancer Institute (NCI)'s linked Surveillance Epidemiology and End Results (SEER)-Medicare database.^24^ The SEER cancer registries provide population-based cancer surveillance for 18 areas that represent ∼30% of the United States.^25^ The SEER-Medicare database combines information from the SEER cancer registries with Medicare claims and enrollment data. The combined data include detailed clinical information about the tumors, demographic characteristics, information on cause of death, and Medicare claims for covered health care services from the time of a person's Medicare eligibility.^24^

### Patients

The study cohort included women age 65 and older diagnosed with stage I or 2 first primary breast cancer between 2004 and 2013 who were enrolled in fee-for-service (FFS) Medicare for at least 12 months before and after diagnosis and underwent surgery (mastectomy or BCS) as part of their initial treatment. We excluded patients 85 years and older, those with atypical histologies, and those diagnosed *via* death certificate or autopsy. We excluded patients whose Medicare ZIP code did not coincide with their SEER registry state, those with unknown ZIP codes, and those for whom driving distances could not be calculated (Supplementary Appendix Table SA1).

### Definition and measurement of key variables

Patient-level demographic variables obtained from Medicare included patient age, race, marital status, dual Medicare-Medicaid eligibility status, and level of comorbidity. Patient-level tumor characteristics obtained from SEER included disease stage, grade, regional node involvement, and hormone receptor status. ZIP-code level demographic variables included median household income and level of rurality. Comorbidity was calculated by using the Charlson Comorbidity Score (NCI 2014 version),^26^ categorized into 0, 1, 2, and 3+. Median household income was categorized into approximate quartiles of <$45,000, $45,000–60,000, $60,000–75,000, and >$75,000. For all demographic and disease variables, missing values were dummy coded into their own category.

Type of surgery, delivery of radiation treatment, and the type of radiation treatment were identified by the relevant Current Procedural Terminology codes^27^ in the patient Medicare claims (Supplementary Appendix Table SA2). We restricted our analysis of radiation completeness to patients who had conventional radiotherapy, intensity-modulated radiation therapy (IMRT), a combination of conventional and IMRT, or brachytherapy. We excluded patients who had alternative forms of radiation (stereotactic body radiation treatment, proton beam radiation, neutron beam radiation, and electronic brachytherapy), and those for whom type of radiation treatment received could not be distinguished from their claims. Incomplete radiation was defined as patients who received less than 15 U of conventional radiotherapy or IMRT. This is a fairly conservative estimate of completion compared with other studies^2–4,28^ and reflects the evolving understanding of the effectiveness of shorter-course regimens,^29^ allowing these courses to be considered complete. Patients with less than 15 U of radiotherapy who died within 60 days of their last documented radiation treatment were not considered to have received an incomplete course of radiation.

### Identification of radiation treatment facilities

Among patients who received radiation, we determined the location where radiation treatment occurred by the claim service facility ZIP code if the radiation claim came from the outpatient file and the provider ZIP code if the radiation claim came from the carrier file. We defined available radiation treatment facilities as radiation facilities where at least one patient in our cohort received radiation treatment.

### Distance calculations

Minimum travel distance was defined as the driving distance between the centroid of the patient's ZIP code and the centroid of the ZIP code of the nearest available facility. The nearest available facility was determined by using the Network Analyst extension of ArcGIS Pro 10.6.1,^30^ and driving distances were calculated by using the Google Maps Distance Matrix Application Programming Interface (API).^31^ Distance to the nearest available radiation facility was categorized into four groups (<10, 10–25, 25–50, and >50 miles).

### Statistical analyses

*Treatment decision model:* In our statistical models, we estimated the association between: (1) distance to radiation facilities and surgical choice (mastectomy vs. BCS); and (2) distance to radiation facilities and receipt of RT among patients receiving BCS. For the model of radiation receipt after BCS, those defined as having incomplete radiation were included in the BCS + RT category, due to sample size constraints and to provide a more conservative estimate of nonreceipt. We also estimated a model comparing those who received incomplete radiation with those receiving a complete course of radiation. All models controlled for patient age, race, marital status, year of diagnosis, county-level household income, dual Medicare-Medicaid eligibility, level of co-morbidity, tumor grade, hormone receptor status, regional node involvement, and SEER registry. To account for geographic variation in mastectomy rates,^32^ standard errors were clustered at the state level. We considered three alternative discrete choice models: (1) two independent logit models, (2) a multinomial logit model, and (3) a bivariate probit model with sample selection. Each of these three specifications makes different assumptions about the relationship between these treatment decisions.^33–35^

Some recent evidence suggests that RT after BCS may not be necessary for certain patients in our study cohort (women aged 70 and above with stage 1, ER+ tumors).^36,37^ As a sensitivity analysis, we eliminated such patients from our analysis of radiation receipt after BCS. As an additional sensitivity analysis, we added a provider fixed effect for the facility where the patient underwent surgery.

### Survival analysis

To understand the survival implications of both travel distance and receipt of guideline-concordant treatment, we used a Cox proportional hazards model to evaluate whether there were survival differences associated with travel distance as well as receipt of guideline-concordant treatment (mastectomy or BCS+RT) versus guideline-discordant treatment (BCS without RT) in our study population.

We used SAS software, version 9.1.4 (SAS Institute, Inc., Cary, NC, USA) for all analyses, and we considered *p* < 0.05 to be statistically significant. The study was reviewed by the Institutional Review Board (IRB) of the University of Minnesota who determined the study was exempt from full review.

## Results

We identified 61,169 patients who met our inclusion criteria (Table 1). We found that 32% of patients were treated with mastectomy, 54% were treated with BCS+RT, 12% were treated with BCS without RT, and 2% were treated with BCS plus incomplete RT. Three-quarters (75%) of patients lived within 10 miles of a radiation facility, whereas 10% lived at least 25 miles from a radiation facility.

**Table 1.** Baseline Patient Demographic and Disease Characteristics

	Distance to the nearest available radiation facility							
	<10 miles		10–25 miles		25–50 miles		>50 miles	
Characteristic	*N*	%	*N*	%	*N*	%	*N*	%
	45,632		9,730		4,786		1,021	
Treatment								
Mastectomy	13,630	30	3,357	35	2,216	46	456	45
BCS + radiation	25,700	56	5,093	52	2,002	42	400	39
BCS + no radiation	5,396	12	1,112	11	502	10	142	14
BCS + incomplete radiation	906	2	168	2	66	1	23	2
Age at diagnosis								
65–69	13,144	29	3,090	32	1,416	30	305	30
70–74	12,847	28	2,843	29	1,383	29	312	31
75–79	11,162	24	2,264	23	1,196	25	226	22
80–84	8,479	19	1,533	16	791	17	178	17
Race								
White	38,778	85	8,907	92	4,390	92	940	92
Black	3,659	8	434	4	311	6	25	2
Other	3,195	7	389	4	85	2	56	5
Marital status								
Single (never married, unmarried, separated, divorced)	8,219	18	1,285	13	617	13	156	15
Married	22,009	48	5,238	54	2,462	51	532	52
Widowed	13,636	30	2,818	29	1,570	33	270	26
Unknown	1,768	4	1,768	18	137	3	63	6
Medicaid dual status								
Yes	6,013	13	1,206	12	784	16	146	14
No	39,619	87	8,524	88	4,002	84	875	86
Disease stage								
1	29,692	65	6,324	65	3,042	64	590	58
2	15,940	35	3,406	35	1,744	36	431	42
Grade								
1	12,139	27	2,703	28	1,303	27	276	27
2	20,404	45	4,183	43	2,052	43	435	43
3+	11,516	25	2,538	26	1,279	27	271	27
Unknown	1,573	3	306	3	152	3	39	4
Regional node positivity								
No nodes positive	34,425	75	7,413	76	3,573	75	748	73
Any nodes positive	7,960	17	1,691	17	862	18	190	19
No nodes tested	3,247	7	3,247	33	351	7	83	8
ER status								
Positive	37,537	82	7,850	81	3,842	80	814	80
Negative	6,427	14	1,423	15	704	15	149	15
Borderline/unknown	1,668	4	457	5	240	5	58	6
Charlson Comorbidity Score								
0	26,809	59	5,787	59	2,800	59	605	59
1	10,662	23	2,322	24	1,111	23	253	25
2	4,144	9	841	9	442	9	83	8
3+	3,268	7	657	7	380	8	67	7
Unknown	749	2	123	1	53	1	13	1

BCS, breast-conserving surgery.

Among radiation patients, 73% received conventional radiotherapy, 12% received a combination of conventional radiotherapy plus IMRT, 9% received brachytherapy, and 4% received IMRT only. Only 0.4% of patients received an alternative type of radiation, and type of radiation could not be distinguished for 0.8% of patients. Frequency of radiation treatment type remained fairly stable over time, with a slight decrease in conventional radiotherapy and a slight increase in conventional radiotherapy plus IMRT or IMRT only. The median conventional radiotherapy patient received 33 radiation treatment units over a period of 50 days, whereas the median brachytherapy patient received 15 radiation treatment units over a period of 8 days (see Supplementary Appendix Table SA3 for full results).

### Treatment model results

Table 2 presents the results of the multivariate logistic regression models. We found our results to be robust to treatment model specification—coefficient estimates across the three models did not differ appreciably in either magnitude or direction. For simplicity and ease of comparison with other studies, we report the results of the two independent logit models throughout the remainder of the article. Coefficient estimates for each of the three models are reported in Supplementary Appendix Table SA4. Increased distance to the nearest radiation facility was associated with increasing odds of mastectomy (*p* < 0.001). Compared with living within 10 miles of a radiation facility, living 10–25 miles away increased the odds of mastectomy by 6% (odds ratio [OR]: 1.06, 95% confidence interval [CI]: 1.01–1.13), whereas living 25–50 miles away increased the odds of mastectomy by 43% (OR: 1.43, 95% CI: 1.30–1.57), and living >50 miles away increased the odds of mastectomy by 48% (OR: 1.48, 95% CI: 1.22–1.79). Other factors significantly associated with increased odds of mastectomy included older age, Other race, being single or widowed, Medicaid dual status, higher levels of comorbidity, higher stage and higher grade tumors, and negative or borderline/unknown ER status. No lymph node testing and higher median household income were associated with decreased odds of mastectomy. Adding a provider fixed effect does somewhat attenuate the association between distance and treatment decisions, but the associations remain significant, particularly among patients living >25 miles from the nearest radiation facility (Supplementary Appendix Table SA5).

**Table 2.** Odds Ratio Estimates of Selected Characteristics on Treatment Decisions

	Odds of mastectomy versus BCS				Odds of no RT versus any RT after BCS^a^				Odds of incomplete RT versus complete RT after BCS			
	OR^b^	95% CI		*p*^c^	OR^b^	95% CI		*p*^c^	OR ^b^	95% CI		*p*^c^
Distance to nearest radiation facility												
<10 miles	REF			<0.0001	REF			<0.0001	REF			0.00
10–25 miles	1.06	0.97	1.17		1.09	1.01	1.16		0.89	0.68	1.16	
25–50 miles	1.43	1.23	1.67		1.23	1.08	1.39		1.15	0.84	1.57	
>50 miles	1.48	1.17	1.86		1.72	1.37	2.15		1.79	1.24	2.60	
Age at diagnosis												
65–69	REF			<0.0001	REF			<0.0001	REF			<0.0001
70–74	1.07	1.00	1.14		1.23	1.15	1.33		1.10	0.94	1.28	
75–79	1.13	1.04	1.22		1.79	1.69	1.90		1.01	0.87	1.17	
80–84	1.30	1.18	1.43		3.12	2.76	3.53		1.35	1.14	1.59	
Race												
White	REF			<0.0001	REF			<0.0001	REF			0.36
Black	0.96	0.87	1.05		1.27	1.14	1.41		1.18	0.82	1.70	
Other	1.56	1.40	1.73		0.88	0.75	1.02		0.93	0.74	1.17	
Marital status												
Married	REF			<0.0001	REF			<0.0001	REF			0.01
Single	1.12	1.07	1.18		1.23	1.12	1.35		0.95	0.82	1.11	
Widowed	1.15	1.11	1.20		1.17	1.10	1.25		0.93	0.82	1.05	
Unknown	1.12	1.04	1.73		1.37	1.07	1.75		1.50	1.01	2.20	
Medicaid dual status												
No	REF			<0.0001	REF			<0.0001	REF			0.43
Yes	1.44	1.39	1.49		1.36	1.23	1.50		1.08	0.89	1.31	
Charlson Comorbidity Score												
0	REF			<0.0001	REF			<0.0001	REF			0.15
1	1.09	1.03	1.15		1.03	0.95	1.12		1.00	0.88	1.13	
2	1.18	1.13	1.24		1.27	1.18	1.36		1.15	0.97	1.37	
3+	1.34	1.25	1.43		1.43	1.33	1.54		1.13	0.96	1.32	
Stage												
1	REF			<0.0001	REF			0.06	REF			0.04
2	2.48	2.34	2.63		1.08	1.00	1.17		0.86	0.75	0.99	
Grade												
1	REF			<0.0001	REF			<0.0001	REF			0.05
2	1.23	1.16	1.30		0.79	0.74	0.85		0.86	0.75	0.99	
3+	1.42	1.34	1.49		0.73	0.66	0.80		0.83	0.67	1.02	
Unknown	1.48	1.38	1.58		0.96	0.81	1.13		0.82	0.57	1.19	
Regional node positivity												
No nodes positive	REF			<0.0001	REF			<0.0001	REF			0.00
Any nodes positive	1.04	0.98	1.10		0.76	0.62	0.93		0.88	0.68	1.14	
No nodes tested	0.34	0.30	0.38		4.47	4.16	4.80		1.18	1.01	1.37	
ER status												
Positive	REF			<0.0001	REF			<0.0001	REF			0.32
Negative	1.21	1.15	1.28		0.89	0.80	0.99		1.07	0.89	1.28	
Borderline/unknown	1.63	1.43	1.85		1.46	1.15	1.85		0.86	0.49	1.53	
Median household income percentile												
<$45,000	REF			0.00	REF			0.37	REF			0.61
$45,000–60,000	0.93	0.85	1.01		0.97	0.90	1.04		1.02	0.85	1.21	
$60,000–75,000	0.92	0.85	0.99		0.99	0.94	1.04		0.96	0.67	1.38	
>$75,000	0.85	0.78	0.92		0.90	0.79	1.02		0.83	0.57	1.21	

Models also control for year of diagnosis and SEER registry from which the data were obtained.

^a^ In this model, BCS+RT includes both complete and incomplete radiation.

^b^ ORs >1 are interpreted as increasing odds of mastectomy (model 1), increasing odds that a BCS patient did not receive RT (model 2), and increasing odds that a BCS patient received incomplete RT (model 3).

^c^ Global *p*-values calculated by using a Type 3 Wald test.

CI, confidence interval; OR, odds ratio; RT, radiation therapy; SEER, Surveillance Epidemiology and End Results.

Similarly, increased distance to the nearest radiation facility was associated with increasing odds that a BCS patient would not receive RT (*p* < 0.001). Compared with living within 10 miles of a radiation facility, living 10–25 miles away increased the odds of no radiation by 9% (OR: 1.09, 95% CI: 0.99–1.19), whereas living 25–50 miles away increased the odds of no radiation by 23% (OR: 1.23, 95% CI: 1.07–1.41), and living >50 miles away increased the odds of no radiation by 72% (OR: 1.72, 95% CI: 1.32–2.23).

Other factors significantly associated with increased odds of failing to receive RT after BCS included older age, Black race, being single or widowed, Medicaid dual status, higher levels of comorbidity, not receiving lymph node testing, and borderline/unknown ER status. The combination of no lymph node testing and unknown ER status suggests that not receiving RT is associated with those receiving less comprehensive care overall. Other race, higher grade and ER-tumors, and lymph node positivity were associated with decreased odds of not receiving RT. Although higher median household income was significantly associated with lower mastectomy rates, it was not associated with rates of RT completion. Restricting our sample to those who would not be eligible for BCS without RT under current guidelines did not impact the results.

Living >50 miles from the nearest radiation facility was also associated with increasing odds of receiving an incomplete course of RT (OR = 1.79, 95% CI: 1.24–2.60), though this association was not observed at distances of 10–25 or 25–50 miles. Age 80–84 years, unknown marital status, and not receiving lymph node testing were significantly associated with receiving an incomplete course of radiation. Stage 2 patients were less likely to receive an incomplete course of radiation than stage 1 patients.

### Survival analysis

Supplementary Appendix Figure SA1 shows the unadjusted relationship between patient travel distance to the nearest radiation facility and survival. Patients living >50 miles from a radiation facility experienced worse survival outcomes compared with patients living <10 miles from a radiation facility (*p* < 0.001). In our multivariable Cox model, this association between patient travel distance and survival is no longer significant. However, in this model, patients receiving guideline-discordant care (BCS without RT) had significantly worse overall (HR: 1.50, 95% CI: 1.42–1.57) and breast-cancer specific survival (HR: 1.44, 95% CI: 1.29–1.60) than those receiving guideline-concordant treatment (Table 3).

**Table 3.** Cox Proportional Hazards Models of Overall and Breast Cancer-Specific 10-Year Survival

	Overall survival				Breast cancer-specific survival			
	HR^a^	95% CI		95% CI	HR ^a^	95% CI		*p*-Value^b^
Treatment choice				<0.0001				<0.0001
Guideline-concordant (mastectomy or BCS + RT)	REF				REF			
Guideline-discordant (BCS + no RT)	1.51	1.41	1.61		1.43	1.30	1.58	
Distance to nearest radiation facility				0.24				0.34
<10 miles	REF				REF			
10–25 miles	1.04	0.99	1.09		1.07	0.97	1.18	
25–50 miles	1.03	0.96	1.11		1.10	0.96	1.25	
>50 miles	1.11	0.97	1.27		1.11	0.86	1.43	
Age at diagnosis				<0.0001				<0.0001
65–69	REF				REF			
70–74	1.34	1.26	1.41		1.07	0.97	1.18	
75–79	1.95	1.85	2.06		1.43	1.30	1.58	
80–84	2.97	2.80	3.14		1.80	1.62	2.00	
Race				<0.0001				0.01
White	REF				REF			
Black	1.31	1.22	1.41		1.05	0.93	1.18	
Other	0.70	0.64	0.77		0.77	1.04	1.28	
Marital status				<0.0001				0.00
Married	REF				REF			
Single	1.15	1.09	1.22		1.15	1.04	1.28	
Widowed	1.16	1.11	1.21		1.16	1.07	1.26	
Unknown	1.08	0.98	1.19		1.01	0.83	1.22	
Medicaid dual status				<0.0001				<0.0001
No	REF				REF			
Yes	1.35	1.29	1.42		1.36	1.23	1.49	
Charlson Comorbidity Score				<0.0001				<0.0001
0	REF				REF			
1	1.65	1.58	1.72		1.23	1.13	1.34	
2	2.24	2.11	2.36		1.37	1.22	1.54	
3+	3.71	3.51	3.92		1.88	1.67	2.11	
Stage				<0.0001				<0.0001
1	REF				REF			
2	1.42	1.35	1.48		2.48	2.28	2.71	
Grade				<0.0001				<0.0001
1	REF				REF			
2	1.14	1.09	1.20		1.58	1.40	1.78	
3+	1.38	1.30	1.45		2.78	2.46	3.14	
Unknown	1.17	1.06	1.29		1.46	1.16	1.82	
Regional node positivity				<0.0001				<0.0001
No nodes positive	REF				REF			
Any nodes positive	1.15	1.08	1.21		1.40	1.29	1.53	
No nodes tested	1.66	1.57	1.76		2.04	1.81	2.29	
ER status				<0.0001				<0.0001
Positive	REF				REF			
Negative	1.34	1.27	1.41		1.91	1.76	2.07	
Borderline/unknown	1.08	1.00	1.16		1.20	1.03	1.40	
Median household income percentile				0.00				0.16
<$45,000	REF				REF			
$45,000–60,000	0.99	0.94	1.04		0.99	0.90	1.09	
$60,000–75,000	0.96	0.91	1.02		0.93	0.83	1.03	
>$75,000	0.90	0.84	0.95		0.89	0.80	1.00	

Models also control for year of diagnosis and SEER registry from which the data were obtained.

^a^ HRs >1 are interpreted as increasing odds of death.

^b^ Global *p*-values calculated by using a Type 3 Wald test.

HR, hazards ratio.

## Discussion

Our results strongly suggest that the distance breast cancer patients must travel to access radiation is associated with both their surgical treatment decision (mastectomy vs. BCS) and their decision to receive recommended radiation after BCS. These findings build on previous studies^14–21,23,38,39^ and add to a growing body of research that recognizes patient travel distance as an important barrier to access in cancer care, particularly among older and more vulnerable populations.^40–42^ Our study also helps to illustrate the mechanism through which observed rural–urban survival disparities may occur—patients living farther from treatment facilities are more likely to receive guideline-discordant treatment, and those receiving guideline-discordant treatment experience worse survival outcomes.^43^

Our study used strong data and innovative methods to improve on previous research in several ways. Using SEER-Medicare linked claims data, we were able to verify patient receipt of RT. To our knowledge, ours is the first study to attempt to characterize and quantify incomplete RT in a population-based study. Our sample included a large and diverse population of Medicare patients across 12 states. We also employed more recently available and sophisticated measures of calculating travel distance that allowed us to compute actual driving distances, rather than rely on great circle or “as the crow flies” measures of distance. Our findings were robust to various specifications of the treatment decision model. This strengthens our confidence in the observed association between travel distance and treatment decisions as well as the findings of previous studies that did not consider alternative model specifications.

In our patient cohort, 12% of patients were treated with BCS without RT and 2% of patients were treated with incomplete RT. This means that 14% of all patients and 20% of BCS patients in this cohort received guideline-discordant care. The proportion of patients receiving guideline-discordant care is similar to those reported in previous studies.^5,8–10^ Our findings suggest that patients receiving incomplete RT make up only a small percentage of breast cancer patients, and that the majority (86%) of patients receiving guideline-discordant care are not receiving any RT. However, those patients living more than 50 miles from a radiation facility are more likely to have incomplete courses of radiation. Using Medicare claims also allowed us to identify the type of radiation patients received. Among RT patients in our cohort, most (91%) received conventional radiotherapy, IMRT, or a combination of the two. Only 9% of patients in our study cohort received brachytherapy; this proportion is similar to other studies of brachytherapy use in the Medicare population.^44^

Although there is ample literature on patient autonomy and shared decision making in breast cancer treatment,^45–50^ the question of whether surgical and radiation decisions are considered separately or in tandem has not been addressed. Numerous decision aids for breast cancer surgical decision making have been developed and tested,^51,52^ whereas a few decision aids exist to assist patients and physicians with radiotherapy decision making.^45,53^

Understanding how clinicians and patients can improve adherence to guideline-concordant treatment is particularly important, given that in our study we continue to see survival differences between those who receive guideline-concordant treatment and those who do not. Existing evidence suggests that surgeon participation in the radiation decision may lead to more guideline-concordant care,^54^ but that many surgeons may have inadequate knowledge of the role of radiation in proper breast cancer management.^55^

Adoption of shorter course radiation treatment regimens is a strategy that could both reduce travel burden and improve treatment adherence among breast cancer patients. Despite the establishment of noninferiority of shorter course hypofractionated RT^13,56,57^ and its recommendation for use among older breast cancer patients,^57^ uptake of this regimen in the United States has been slow.^58^ Similarly, omission of radiation after BCS for eligible elderly breast cancer patients has also been slow to disseminate after the release of updated guidelines.^59,60^ Provider-side financial incentives in FFS Medicare, in which additional fractions are tied to additional reimbursement, may be driving this slow uptake.^58,61^ Adoption of value-based payment models could help accelerate adoption.

Although brachytherapy in older breast cancer patients has been associated with a higher risk of complications and subsequent mastectomy compared with conventional radiotherapy,^44^ brachytherapy does significantly reduce the median time period over which radiation treatment occurs (from 50 to 8 days in our study cohort), and it may be an attractive alternative for patients for whom travel burden is the primary barrier to completing RT. Ongoing clinical trial research^62^ suggests that safer, more effective brachytherapy techniques may present an alternative treatment option for elderly breast cancer patients in future.

Apart from modifying treatment regimens, clinicians and policymakers should explore service delivery and social support models that reduce travel burden among breast cancer patients and thereby reduce its salience on their treatment decisions. Lodging,^63^ transportation support,^64,65^ and transportation reimbursement^66^ models could be adapted or expanded.

This study has several limitations. First, our study cohort is restricted to women age 65 and over enrolled in FFS Medicare; therefore, the results may not be generalizable to younger women or those enrolled in Medicare Advantage plans. An important strength of using FFS Medicare data is that it precludes the potential confounding effect of insurance status. Because virtually every provider accepts FFS Medicare, we can say with some certainty that if a radiation facility or reconstruction provider exists in a geographic area covered by the SEER program, a patient in our study cohort would have coverage to use it. This would not necessarily be true among commercially insured patients, for whom network participation would be a major consideration. Second, our measure of available radiation facilities relies on observation of the facility in the SEER-Medicare claims data. This may lead us to either overestimate or underestimate distance to the nearest available facility. Distance to the nearest available facility would be overestimated if the nearest available facility to a patient was not visited by any other patient in our cohort. To minimize this risk, we excluded patients coming from non-SEER registry states. Distance to the nearest available facility would be underestimated if we included in our analysis a facility that is not actually providing radiation treatment services; for example, if the zip code on the claim was not the actual zip code where the treatment was provided or if a satellite radiation facility billed through a centralized health care system billing address.

In the event that this occurred, our analysis would provide a conservative estimate of distance to the nearest available facility. Third, we do not consider the availability of other providers, such as plastic surgeons able to perform breast reconstruction, which may affect a patient's decision of whether or not to undergo a mastectomy. In spite of these limitations, our study contributes valuable new information on the role of patient travel distance on treatment decisions and outcomes among older breast cancer patients across the United States.

## Conclusion

Travel distance to radiation facilities is associated with both the choice of surgical treatment and the decision of whether to undergo RT among breast cancer patients in the Medicare population. Patients living farther from radiation facilities were more likely to be treated with mastectomy compared with BCS and less likely to complete recommended RT after BCS. Patients treated with BCS without RT experienced worse breast-cancer specific and overall survival outcomes compared with patients treated with guideline-concordant treatment (mastectomy or BCS+RT). Thus, rural–urban survival disparities among cancer patients may be, in part, attributable to differential access to guideline-concordant treatment resulting from excess travel burden among rural patients. Clinicians, policymakers, and patient advocates should explore social support and service delivery models at reducing travel burden and improving guideline-concordant treatment among this patient population.

## Supplementary Material

Supplemental data

Supplemental data

Supplemental data

Supplemental data

Supplemental data

Supplemental data

## Supplementary Data

Supplementary Appendix Figure SA1

Supplementary Appendix Table SA1

Supplementary Appendix Table SA2

Supplementary Appendix Table SA3

Supplementary Appendix Table SA4

Supplementary Appendix Table SA5

## Abbreviations Used

BCS: breast-conserving surgery
CI: confidence interval
FFS: fee-for-service
HR: hazards ratio
IMRT: intensity-modulated radiation therapy
NCI: National Cancer Institute
OR: odds ratio
RT: radiation therapy
SEER: Surveillance Epidemiology and End Results
