# Supplementary material for: Travel, Treatment Choice, and Survival Among Breast Cancer Patients: A Population-Based Analysis
Source: Womens Health Rep (New Rochelle). 2021 Jan 11;2(1):1–10. doi: 10.1089/whr.2020.0094 (PMC7957915; doi:10.1089/whr.2020.0094)
Supplement: Supplemental data [file Supp_TableS1.docx]

**Appendix Table 1: Study Cohort Selection**

| **Step** | **Total Cohort** | **Number Lost** | **Percent Lost** |
| --- | --- | --- | --- |
| Total breast cancer cases in PEDSF diagnosed between 2004-2013: | 387,484 |  |  |
| Restrict to female: | 384,065 | 3,419 | 0.88% |
| Restrict to age >= 65 at diagnosis: | 246,548 | 137,517 | 35.81% |
| Restrict to first primary (sequence no. 00 or 01) or secondary if first cancer not breast: | 224,717 | 21,831 | 8.85% |
| Restrict to not diagnosed at death certificate or autopsy: | 222,830 | 1,887 | 0.84% |
| Restrict to typical histology (8000, 8010, 8140, 8230, 8480, 8500, 8504, 8507, 8510, 8521, 8522, 8523, 8524, 8575) | 187,218 | 35,612 | 15.98% |
| **Restrict to those without stage IV disease:** | 168,046 | 19,172 | 10.24% |
| Restrict to those who had surgery: | 161,515 | 6,531 | 3.89% |
| Restrict to known month of diagnosis: | 161,173 | 342 | 0.21% |
| Restrict to HMO and entitlement status: | 97,552 | 63,621 | 39.47% |
| Restrict to patients living in SEER-registry states: | 96,709 | 843 | 0.86% |
| Restrict to patients whose driving distances could be calculated: | 96,566 | 143 | 0.15% |
| **Restrict to patients age 65-84 years old with stage I or II disease:** | 61,169 | 35,397 | 36.66% |
