# Supplementary material for: Travel, Treatment Choice, and Survival Among Breast Cancer Patients: A Population-Based Analysis
Source: Womens Health Rep (New Rochelle). 2021 Jan 11;2(1):1–10. doi: 10.1089/whr.2020.0094 (PMC7957915; doi:10.1089/whr.2020.0094)
Supplement: Supplemental data [file Supp_TableS2.docx]

**Appendix Table 2: CPT Codes Used to Identify Treatments**

| **Treatment** | **ICD-9 Procedure Codes** | **CPT Codes** |
| --- | --- | --- |
| Mastectomy | 8533 - 8548 | 19180, 19182, 19200, 19220, 19240, 19303, 19304, 19305, 19306, 19307 |
| Breast-Conserving Surgery | 8520, 8521, 8522, 8523, 8525 | 19110, 19120, 19125, 19126, 19160, 19162, 19301, 19302 |
| Delivery of Conventional Radiotherapy Treatment |  | 77401 - 77416 |
| Delivery of IMRT Treatment |  | 77418, 77385 - 77387 |
| Delivery of Brachytherapy Treatment |  | 77750 - 77799 |
| Delivery of Other Types of Radiation Treatment (Neutron beam, Proton beam, SBRT, SRS, Electronic brachytherapy) |  | 77371 – 77373, 77422 – 77425, 77520 – 77525, 0182T, 0394T, 0395T, G0173, G0174, G0243, G0251, G0338, G0339, G0340 |
| Radiation-Associated Codes (Treatment planning, blocking, dosimetry, etc.) |  | 77014, 77261 - 77263, 77280 - 77290, 77295, 77300, 77301, 77307, 77316, 77317, 77318, 77321, 77331, 77333, 77334, 77336, 77338, 77379, 77417, 77427, 77431, 77435, 77469, 77470, 77790 |
