# Supplementary material for: Travel, Treatment Choice, and Survival Among Breast Cancer Patients: A Population-Based Analysis
Source: Womens Health Rep (New Rochelle). 2021 Jan 11;2(1):1–10. doi: 10.1089/whr.2020.0094 (PMC7957915; doi:10.1089/whr.2020.0094)
Supplement: Supplemental data [file Supp_TableS3.docx]

**Appendix Table 3: Type of Radiation and Length of Treatment**

|  | **Number** | **Percent** | **Median units** | **IQR units** | **Median timeframe (days)** | **IQR timeframe** |
| --- | --- | --- | --- | --- | --- | --- |
| Total BCS+RT cohort | 34,358 | 100.0% |  |  |  |  |
| Conventional radiotherapy | 25,220 | 73.4% | 33 | (30 - 33) | 50 | (45 - 58) |
| Conventional radiotherapy + IMRT | 4,189 | 12.2% | 33 | (33 - 34) | 52 | (48 - 60) |
| Brachytherapy | 3,164 | 9.2% | 15 | (10 - 20) | 8 | (6 - 13) |
| IMRT only | 1,356 | 3.9% | 28 | (16 - 33) | 44 | (29 - 51) |
| Other types of RT: SBRT, Neutron beam, Proton, electronic brachytherapy | 146 | 0.4% |  |  |  |  |
| Multiple types/unable to distinguish | 283 | 0.8% |  |  |  |  |
