# Supplementary material for: Travel, Treatment Choice, and Survival Among Breast Cancer Patients: A Population-Based Analysis
Source: Womens Health Rep (New Rochelle). 2021 Jan 11;2(1):1–10. doi: 10.1089/whr.2020.0094 (PMC7957915; doi:10.1089/whr.2020.0094)
Supplement: Supplemental data [file Supp_TableS4.docx]

**Appendix Table 4: Coefficient Estimates of the Three Specifications of the Treatment Decision Model***

|  | **Two Independent Regressions** | | | | **Multinomial Logit** | | | | **Bivariate Probit** | | | |
| --- | --- | --- | --- | --- | --- | --- | --- | --- | --- | --- | --- | --- |
|  | *Mastectomy* | | *No RT Following BCS* | | *Mastectomy* | | *No RT Following BCS* | | *Mastectomy* | | *No RT Following BCS* | |
|  | Estimate | Std Err | Estimate | Std Err | Estimate | Std Err | Estimate | Std Err | Estimate | Std Err | Estimate | Std Err |
| *Distance to Nearest Radiation Facility* |  |  |  |  |  |  |  |  |  |  |  |  |
| < 10 miles | REF |  | REF |  | REF |  | REF |  | REF |  | REF |  |
| 10-25 miles | 0.06 | 0.05 | 0.08 | 0.03 | **0.08** | 0.03 | **0.08** | 0.04 | **0.04** | 0.02 | **0.05** | 0.02 |
| 25-50 miles | **0.36** | 0.08 | **0.20** | 0.07 | **0.39** | 0.04 | **0.18** | 0.06 | **0.22** | 0.02 | **0.12** | 0.03 |
| > 50 miles | **0.39** | 0.12 | **0.54** | 0.11 | **0.50** | 0.07 | **0.54** | 0.11 | **0.23** | 0.04 | **0.32** | 0.06 |
| *Age at Diagnosis* |  |  |  |  |  |  |  |  |  |  |  |  |
| 65-69 | REF |  | REF |  | REF |  | REF |  | REF |  | REF |  |
| 70-74 | **0.07** | 0.03 | **0.21** | 0.04 | **0.09** | 0.03 | **0.21** | 0.04 | **0.04** | 0.01 | **0.11** | 0.02 |
| 75-79 | **0.12** | 0.04 | **0.58** | 0.03 | **0.20** | 0.03 | **0.59** | 0.04 | **0.07** | 0.02 | **0.31** | 0.02 |
| 80-84 | **0.26** | 0.05 | **1.14** | 0.06 | **0.47** | 0.03 | **1.14** | 0.04 | **0.15** | 0.02 | **0.64** | 0.02 |
| *Race* |  |  |  |  |  |  |  |  |  |  |  |  |
| White | REF |  | REF |  | REF |  | REF |  | REF |  | REF |  |
| Black | -0.04 | 0.05 | **0.24** | 0.05 | 0.00 | 0.04 | **0.23** | 0.06 | -0.03 | 0.02 | **0.14** | 0.03 |
| Other | **0.45** | 0.05 | -0.13 | 0.08 | **0.42** | 0.04 | **-0.13** | 0.07 | **0.26** | 0.02 | -0.05 | 0.04 |
| *Marital Status* |  |  |  |  |  |  |  |  |  |  |  |  |
| Married | REF |  | REF |  | REF |  | REF |  | REF |  | REF |  |
| Single | **0.11** | 0.03 | **0.21** | 0.05 | **0.14** | 0.03 | **0.21** | 0.04 | **0.07** | 0.02 | **0.12** | 0.02 |
| Widowed | **0.14** | 0.02 | **0.16** | 0.03 | **0.17** | 0.02 | **0.16** | 0.03 | **0.09** | 0.01 | **0.09** | 0.02 |
| Unknown | **0.11** | 0.03 | **0.31** | 0.13 | **0.16** | 0.05 | **0.31** | 0.07 | **0.07** | 0.03 | **0.17** | 0.04 |
| *Medicaid Dual Status* |  |  |  |  |  |  |  |  |  |  |  |  |
| No | REF |  | REF |  | REF |  | REF |  | REF |  | REF |  |
| Yes | **0.36** | 0.02 | **0.31** | 0.05 | **0.42** | 0.03 | **0.30** | 0.04 | **0.22** | 0.02 | **0.18** | 0.03 |
| *Charlson Comorbidity Score* |  |  |  |  |  |  |  |  |  |  |  |  |
| 0 | REF |  | REF |  | REF |  | REF |  | REF |  | REF |  |
| 1 | **0.08** | 0.03 | 0.03 | 0.04 | **0.09** | 0.02 | 0.04 | 0.03 | **0.05** | 0.01 | 0.02 | 0.02 |
| 2 | **0.17** | 0.02 | **0.24** | 0.04 | **0.21** | 0.03 | **0.25** | 0.05 | **0.10** | 0.02 | **0.14** | 0.03 |
| 3+ | **0.29** | 0.04 | **0.36** | 0.04 | **0.37** | 0.04 | **0.37** | 0.05 | **0.17** | 0.02 | **0.22** | 0.03 |
| *Stage* |  |  |  |  |  |  |  |  |  |  |  |  |
| 1 | REF |  | REF |  | REF |  | REF |  | REF |  | REF |  |
| 2 | **0.91** | 0.03 | 0.08 | 0.04 | 0.92 | 0.03 | 0.06 | 0.04 | **0.55** | 0.02 | **0.07** | 0.02 |
| *Grade* |  |  |  |  |  |  |  |  |  |  |  |  |
| 1 | REF |  | REF |  | REF |  | REF |  | REF |  | REF |  |
| 2 | **0.21** | 0.03 | **-0.23** | 0.03 | **0.17** | 0.02 | **-0.23** | 0.03 | **0.12** | 0.01 | **-0.12** | 0.02 |
| 3+ | **0.35** | 0.03 | **-0.32** | 0.05 | **0.30** | 0.03 | **-0.30** | 0.04 | **0.21** | 0.02 | **-0.16** | 0.02 |
| Unknown | **0.39** | 0.03 | -0.04 | 0.08 | **0.38** | 0.06 | -0.03 | 0.07 | **0.23** | 0.03 | -0.01 | 0.04 |
| *Regional Node Positivity* |  |  |  |  |  |  |  |  |  |  |  |  |
| No Nodes Positive | REF |  | REF |  | REF |  | REF |  | REF |  | REF |  |
| Any Nodes Positive | 0.04 | 0.03 | **-0.28** | 0.10 | 0.00 | 0.03 | **-0.27** | 0.06 | 0.03 | 0.02 | **-0.14** | 0.03 |
| No Nodes Tested | **-1.09** | 0.06 | **1.50** | 0.04 | **-0.56** | 0.05 | **1.51** | 0.04 | **-0.62** | 0.03 | **0.87** | 0.02 |
| *ER Status* |  |  |  |  |  |  |  |  |  |  |  |  |
| Positive | REF |  | REF |  | REF |  | REF |  | REF |  | REF |  |
| Negative | **0.19** | 0.03 | **-0.12** | 0.05 | **0.18** | 0.03 | **-0.13** | 0.05 | **0.12** | 0.02 | **-0.06** | 0.03 |
| Borderline/Unknown | **0.49** | 0.07 | **0.38** | 0.12 | **0.57** | 0.05 | **0.39** | 0.07 | **0.30** | 0.03 | **0.21** | 0.04 |
| *Median Household Income Percentile* |  |  |  |  |  |  |  |  |  |  |  |  |
| <$45,000 | REF |  | REF |  | REF |  | REF |  | REF |  | REF |  |
| $45,000 - 60,000 | **-0.07** | 0.04 | -0.03 | 0.04 | **-0.08** | 0.03 | -0.04 | 0.04 | **-0.05** | 0.02 | -0.02 | 0.02 |
| $60,000 - 75,000 | **-0.08** | 0.04 | -0.01 | 0.02 | **-0.09** | 0.03 | -0.02 | 0.04 | **-0.05** | 0.02 | -0.01 | 0.02 |
| >$75,000 | **-0.16** | 0.04 | -0.11 | 0.06 | **-0.18** | 0.03 | -0.11 | 0.05 | **-0.10** | 0.02 | **-0.06** | 0.03 |

*Models also control for year of diagnosis and SEER registry from which the data were obtained.

^†^Coefficient estimates in **bold** are significant at the p<0.05 level. Models also control for SEER registry and year of diagnosis.
