# Supplementary material for: Travel, Treatment Choice, and Survival Among Breast Cancer Patients: A Population-Based Analysis
Source: Womens Health Rep (New Rochelle). 2021 Jan 11;2(1):1–10. doi: 10.1089/whr.2020.0094 (PMC7957915; doi:10.1089/whr.2020.0094)
Supplement: Supplemental data [file Supp_TableS5.docx]

**Appendix Table 5: Sensitivity Results of Model with Provider Fixed Effect**

| **Main Model - No Provider Effects** | | | | | | | | |
| --- | --- | --- | --- | --- | --- | --- | --- | --- |
|  | ***Mastectomy*** | | |  | ***No RT Following BCS*** | | | |
|  | **OR** | **95% CI** | | **P-Value** | **OR** | **95% CI** | | **P-Value** |
| ***Distance to Nearest Radiation Facility*** |  |  |  |  |  |  |  |  |
| **< 10 miles** | **REF** |  |  |  | **REF** |  |  |  |
| **10-25 miles** | **1.06** | **0.97** | **1.17** | **0.2087** | **1.09** | **1.01** | **1.16** | **0.0188** |
| **25-50 miles** | **1.43** | **1.23** | **1.67** | **<.0001** | **1.23** | **1.08** | **1.39** | **0.0017** |
| **> 50 miles** | **1.48** | **1.17** | **1.86** | **0.0009** | **1.72** | **1.37** | **2.15** | **<.0001** |
|  |  |  |  |  |  |  |  |  |
|  |  |  |  |  |  |  |  |  |
| **Sensitivity Analysis: Model with Provider Fixed Effects** | | | | | | | | |
|  | ***Mastectomy*** | | |  | ***No RT Following BCS*** | | | |
|  | **OR** | **95% CI** | | **P-Value** | **OR** | **95% CI** | | **P-Value** |
| ***Distance to Nearest Radiation Facility*** |  |  |  |  |  |  |  |  |
| **< 10 miles** | **REF** |  |  |  | **REF** |  |  |  |
| **10-25 miles** | **1.01** | **0.95** | **1.07** | **0.8394** | **1.07** | **0.98** | **1.16** | **0.1590** |
| **25-50 miles** | **1.19** | **1.09** | **1.30** | **0.0001** | **1.17** | **1.01** | **1.35** | **0.0327** |
| **> 50 miles** | **1.37** | **1.14** | **1.64** | **0.0006** | **1.65** | **1.26** | **2.18** | **0.0003** |
