# Supplementary material for: Travel, Treatment Choice, and Survival Among Breast Cancer Patients: A Population-Based Analysis
Source: Womens Health Rep (New Rochelle). 2021 Jan 11;2(1):1–10. doi: 10.1089/whr.2020.0094 (PMC7957915; doi:10.1089/whr.2020.0094)
Supplement: Supplemental data [file Supp_FigureS1.docx]

**
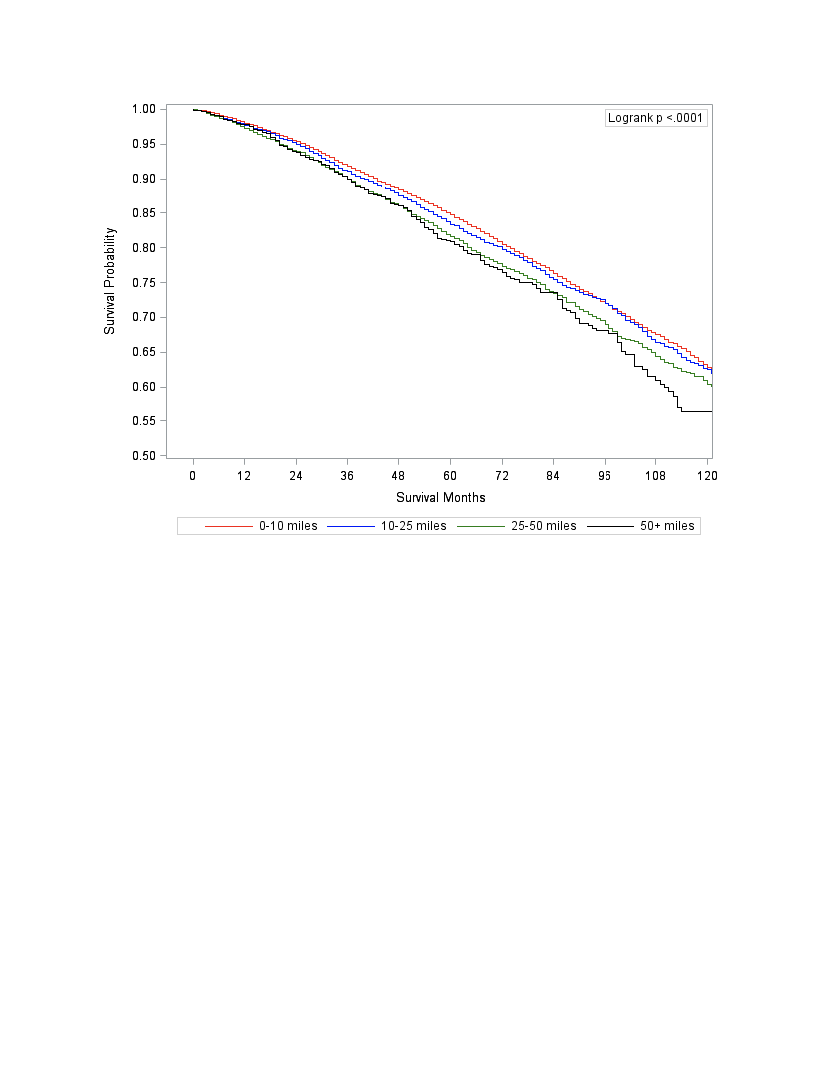
**

**Appendix Figure 1: Unadjusted Relationship between Patient Travel Distance and 10-Year Overall Survival**
